# Significance of M2 macrophage in tubulointerstitial disease secondary to primary Sjogren's disease

**DOI:** 10.1080/0886022X.2018.1518242

**Published:** 2018-11-05

**Authors:** Jun Li, Ya-Fen Yu, Chang-Hua Liu, Cui-Mei Wang

**Affiliations:** a Wuxi School of Medicine, Jiangnan University, Wuxi, China;; b Department of Nephrology, The Affiliated Hospital of Jiangnan University, Wuxi, China;; c Department of Nephrology, Clinical Medical College, Yangzhou University, Yangzhou, China

**Keywords:** M2 macrophage, HIF-1α, primary Sjogren's disease, tubulointerstitial disease, clinicopathologic significance

## Abstract

**Objective:** M2 Macrophages could improve tubulointerstitial disease in animal models. HIF-1αpromotes macrophage polarization and is involved in tubular injury. The study aims to observe the clinicopathologic significance of M2 macrophage and HIF-1α in tubulointerstitial injury secondary to primary Sjogren's disease.

**Methods:** Renal tissue samples from patients with tubulointerstitial disease secondary to primary Sjogren's disease (SS, *n* = 10), chronic tubulointerstitial nephritis secondary to drug (CIN, *n* = 8) were included in this study. The expression of CD163, CD68 and HIF-1α were examined by immunohistochemistry or immunofluorescence.

**Results:** (1) Renal involvement was the first manifestation in seven of ten (7/10) patients with pSS, including proteinuria, renal dysfunction, renal tubular acidosis and multiple renal stone; and two patient had intractable hypokalemia. (2) There were numerous CD163- positive cells and CD68- positive cells infiltration in tubulointerstitial injury of pSS, especially in patients with hypokalemia. CD163 positive cells and HIF-1αwere mainly expressed in acute tubulointerstitial injury of pSS, which positively correlated to N-acetyl-β-D-glucosaminidase and β2-microglobulin. (3) Compared with CIN, patients with pSS had higher serum globulin level, erythrocyte sedimentation rate (ESR) and lower urinary osmotic pressure. (4) During follow-up of one year, six patients with pSS and acute tubular injury acquired improved renal function on therapy of steroid and total glucosides of peony. The remaining four patients with pSS had stable renal function.

**Conclusion:** M2 macrophages are involved in acute tubular injury in patients with primary Sjogren's disease. Early intervention can improve renal function of tubulointerstitial injury secondary to primary Sjogren's disease.

## Introduction

Primary Sjogren's syndrome (pSS) is a chronic autoimmune disease characterized by macrophage infiltration of exocrine glands [1,2]. Greenwell-Wild also recognized a plethora of expression level of macrophage-linked gene in severe lesions of salivary glands of pSS patients [3]. Macrophage infiltration is also a universal feature of glomerular injury in both human and experimental diseases [4,5]. Macrophages are divided into the M1 and M2 subtypes according to the expression of different surface molecules and transcription factors. M2 macrophages have anti-inflammatory and tissue repair properties. One characteristic of M2 macrophages is increased CD163 expression. [6,7]. However, there is still uncertainty about the renal expression of M2 macrophages in patients with tubulointerstitial disease secondary to pSS, the difference of renal macrophage distribution between tubulointerstitial disease secondary to pSS and other etiologies. A study reported that Myeloid HIF-1α attenuates the progression of renal fibrosis in murine obstructive nephropathy [8]. Myeloid HIF-1αpromotes phagocytosis and macrophage polarization to M1 phenotype. This study aims to investigate the immunolocalization and clinical significance of CD163-positive macrophages and HIF-1α in patients with tubulointerstitial disease secondary to pSS. Meanwhile, we also analyzed the difference in the expression of CD163 between tubulointerstitial disease secondary to pSS and drug-associated chronic interstitial nephritis.

## Subjects and methods

### Subjects

Formalin-fixed, paraffin-embedded renal tissues were obtained from patients with pSS (*n* = 10), drug-associated chronic interstitial nephritis (CIN, *n* = 8, the drugs includes proton pump inhibitor and Chinese herbs). Renal tissues samples of minimal change disease patients (MCD, *n* = 5), and those obtained from normal control kidneys (*n* = 3) were used as negative controls. Morning urine samples before renal biopsy were collected for the detection of urinary osmotic pressure, urinary NAG and urinary β2- microglobulin. All patients with pSS fulfilled the international classification criteria for Sjögren’s syndrome (2012 American College of Rheumatology classification criteria for Sjögren’s syndrome) [9]. Exclusion criteria were the presence of malignancy, acute inflammation and sepsis. All the patients agreed and signed the informed consent. This study was performed in accordance with the Declaration of Helsinki. This study obtained the permission of the Ethics Committee of the affiliated hospital of Jiangnan University and the Clinical Medical College of Yangzhou University.

## Methods

### Immunohistochemical detection of CD163, CD68 and HIF-1α

After deparaffinization and rehydration, thin sections (2 μm) of the renal biopsies were incubated in 3% (vol/vol) H_2_O_2_ in methanol for 10 min. Heat-induced epitope retrieval was performed in 10 mmol/L sodium citrate buffer (pH 6.0) in a pressure cooker at 100 °C for 2 min. After the incubation of 1% (wt/vol) bovine serum albumin (BSA), mouse antibodies against CD163 (1:200, ab156769; Abcam, Cambridge, MA), or CD68 (1:50, ZM-0464; ZSGB-BIO, Beijing, China), or rabbit antibodies against HIF-1 α (1:100, ZA0552, ZSGB-BIO, Beijing, China) were used as the primary antibody separately for overnight incubation at 4 °C in a humidified chamber. Anti-mouse and anti-rabbit antibodies (ready to use, GK500705; GENETECH, DAKO, GK500705.Uscnlife) were then used as the secondary antibody for 40-min incubation at 37 °C. The chromogenic reaction was conducted after washing the slides 3 times in PBST (1 × PBS/0.1% Tween 20). Next, hematoxylin and periodic-acid–Schiff staining were performed to counterstain the slides. Negative controls for all antibodies were incubated with preimmune mouse or rabbit serum to prevent false immunostaining.

### Immunofluorescence Co-localization detection of CD163 and CD68

Dual staining was performed to detect the co-localization of CD163 and CD68. The paraffin sections were submerged in 10 mmol/L sodium citrate buffer (pH 6.0) and heated for antigen retrieval at 100 °C for 2 min. Mouse anti-CD68 monoclonal antibody (1:50, ZM-0464; ZSGB-BIO, Beijing, China) and rabbit anti-CD163 polyclonal antibody (1:200, ab-100909; Abcam) were used as the primary antibody for overnight incubation at 4 °C. Then rhodamine (TRITC)-conjugated goat-anti-rabbit IgG (1:50; Jackson Immuno Research Inc., Baltimore, MD) and FITC conjugated goat–anti-mouse IgG (1:50; Jackson Immuno Research Inc) were used as the secondary antibody for 40-min incubation at 37 °C and keep in dark place. The slides were washed 3 times in PBS, 5 min each. After adding a drop of Mounting Medium, the sections were covered with coverslips.

### Immunohistochemical analysis of kidney tissue samples

The slides were evaluated by the attending pathologist who was blinded to patient’s clinical data. To count the number of Immunohistochemical stain positive cells, ten fields were examined and the results were presented as the mean ± SD.

### Statistical analysis

Kruskal–Wallis test was used to analyze the difference of clinical indices and positive cells counts among three groups. Mann–Whitney *U*-test was used to compare the clinical indices and positive cells counts between two groups. Spearman test was conducted for correlation analysis of clinical indices. Statistical significance of tests was indicated when *p* values were <.05.

## Results

### Clinical and pathologic data on patients before renal biopsy

Renal involvement was the first manifestation in 7/10 patients with pSS, including proteinuria, renal dysfunction, renal tubular acidosis and multiple renal stone; and two patients had intractable hypokalemia. Glomerulonephritis was observed in two patients with pSS, including mesangial proliferative nephritis (*n* = 1) and focal glomerulosclerosis (*n* = 1). As noted in Table 1, patients with pSS had higher serum globulin level, erythrocyte sedimentation rate (ESR), urinary N-acetyl-β-D-glucosaminidase (NAG) and lower urinary osmotic pressure in comparison with CIN. There was no difference in serum albumin, proteinuria and eGFR between pSS and CIN.

**Table 1.** Clinical data of patients before renal biopsy.

	sex (male/female)	age (y)	Serum albumin (g/l)	Serum globulin (g/l)	eGFR (ml/min)	urinary osmotic pressure (mOsm/l)	Urinary protein (g/d)	Urinary NAG (U/gCr)
pSS	4/6	47 ± 11	28 ± 4	37 ± 5*	65 ± 28	450 ± 82*	1.8 ± 1.6	22 ± 12
CIN	6/2	40 ± 11	26 ± 6	30 ± 5	70 ± 34	644 ± 177	1.0 ± 0.9	12 ± 5
MCD	3/2	22 ± 3	16 ± 3*	20 ± 4	101 ± 8	780 ± 52	5.6 ± 1.7	0

pSS: primary sjogren's disease; CIN: chronic tubulointerstitial nephritis；MCD: minimal change disease; eGFR: estimated glomerular filtration rate; NAG: N-acetyl-β-D-glucosaminidase. *p < .05.

**Table 2.** CD163-Positive and CD68-positive cell counts in the tubulointerstitium lesions.

	CD163 (tubulointerstitium/HPF)	CD68 (tubulointerstitium/HPF)
pSS	24 ± 6	35 ± 5
CIN	10 ± 3**	23 ± 7*
MCD	3 ± 1	4 ± 2

pSS: primary sjogren's disease; CIN: chronic tubulointerstitial nephritis; MCD: minimal change disease; eGFR: estimated glomerular filtration rate; NAG: N-acetyl-β-D-glucosaminidase. *p < .05; **p < .01.

**Table 3.** Correlation of CD163-positive cells and CD68-positive cells counts with clinical indices.

*r*	CD163/	CD68/
*p*	tubulointerstitium	tubulointerstitium
eGFR	–0.171	–0.700
	0.527	0.004**
urinary osmotic pressure	–0.497	–0.725
	0.05*	0.002**
Urinary NAG	0.522	0.444
	0.038*	0.097
Urinary β2- microglobulin	0.640	0.323
	0.008**	0.241

eGFR: estimated glomerular filtration rate; NAG: N-acetyl-β-D-glucosaminidase; *p < .05; **p < .01.

### Expression of CD163, HIF-1α and CD68 in the negative controls

**Figure 1.**
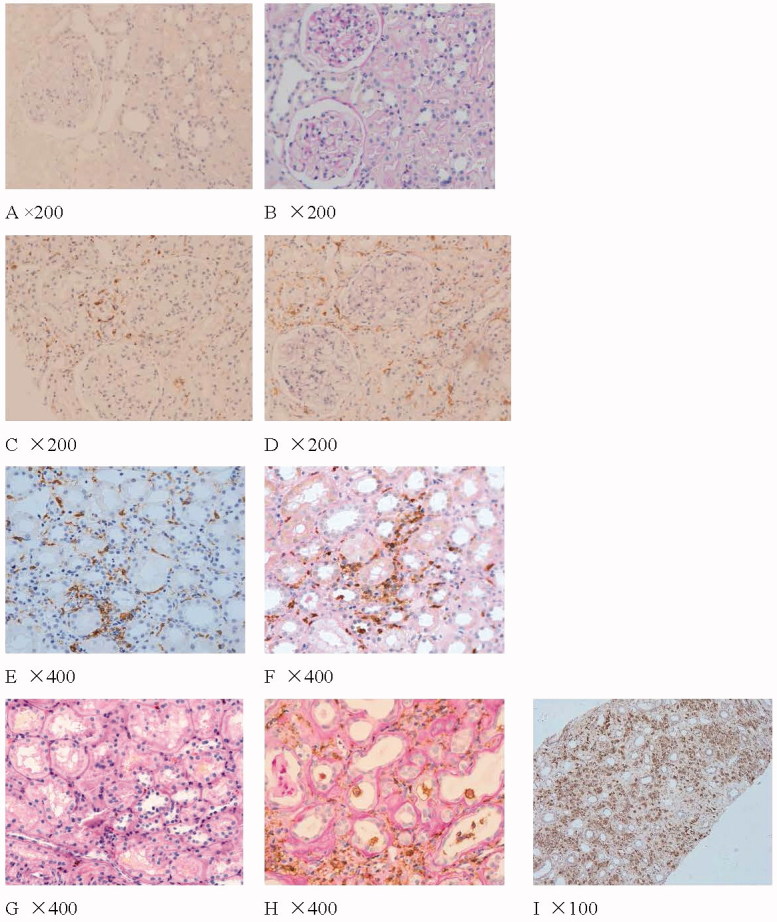
Figure A, C, E, G shows the expression of CD163 in normal renal tissue, pSS, pSS and CIN separately. Figure D, H shows the expression of CD68 in pSS and CIN. Figure B, F shows the expression of HIF1α- in normal renal tissue and pSS. Figure 1 shows the expression of CD68 in pSS with hypokalemia (Immunohistochemistry results, brown). Figure C, D shows the expression of CD163 and CD68 in the same patients; Through the stains of PAS, we can see the acute tubulointerstitial injury. Part of CD163 positive cells also expresses CD68. Figure E, F shows the expression of CD163 and HIF-1α in tubulointerstitial lesions of pSS. CD163 and HIF-1α were mainly observed in acute tubulointerstitial injury of pSS. Figure G shows the expression of fewer CD163 in tubulointerstitial lesions of CIN. Figure H shows the expression of CD68 in chronic tubulointerstitial lesions (PAS stains shows thick tubular basement membrane and shrink tubular cells) of CIN.

As noted in Figure 1 and Table 2, CD68 was occasionally expressed in the tubulointerstitial tissue of normal kidney specimens, and CD163 and HIF-1α staining was negative. In the negative controls (preimmune mouse or rabbit serum were used instead of the primary antibody to examine false immunostaining), CD163, CD68 and HIF-1α staining were all negative. In the MCD samples, CD163 and CD68 were occasionally expressed in tubulointerstitial tissue, HIF-1α staining was negative.

### Expression of CD163 and HIF-1α in the glomeruli and tubulointerstitium of pSS and CIN patients

**Figure 2.**
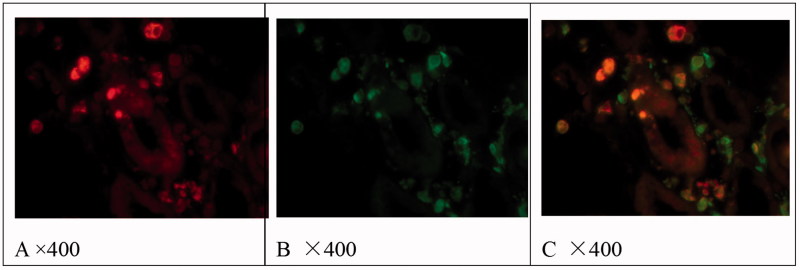
Localization of CD68 (B) and CD163 (A) and co-localization of CD68 and CD163 (C) in tubulointerstitial lesions of pSS. Immunofluorescence results.

As shown in Figures 1 and 2, in pSS and CIN, CD163 and CD68 were occasionally observed in glomeruli. There were numerous CD68-positive cells infiltration and few CD163-positive cells infiltration in tubulointerstitial injury of CIN. There were more CD163 and CD68 positive cells infiltration in tubulointerstitial injury of pSS, especially in patients with hypokalemia. CD68 positive cells were expressed around thickened Bowman's capsule, chronic tubulointerstitial injury and proteinuria casts. Both CD163 and CD68 positive cells and HIF-1α were mainly observed in acute tubulointerstitial injury of pSS.

There was also scattered expression of HIF-1α in the chronic tubular injury of pSS and CIN, which was no significant difference.

### Correlation of the count of CD163-positive cells with clinical index

As noted in Table 3, there was a negative correlation between the number of CD68-positive cells in tubulointerstitial lesions and eGFR (estimated glomerular filtration rate, *r* = –0.700, *p* = .004). There was no correlation between the number of CD163-positive cells in tubulointerstitial lesions and eGFR. CD163 positive cells were positively correlated to both N-acetyl-β-D-glucosaminidase (NAG, *r* = 0.627, *p* = .012) and β2-microglobulin (*r* = 0.602, *p* = .018). The number of CD163-positive cells and CD68-positive cells both negatively correlated to the urinary osmotic pressure (*r* = –0.497, *p* = .05; *r* = –0.725, *p* = .002, separately).

## Discussion

Current data suggest that M2 macrophages are involved in tissue repair in rhabdomyolysis-induced acute kidney injury, adriamycin nephropathy, acute tubule interstitial nephritis, ischemia-reperfusion injury and unilateral ureteral obstruction (UUO) animal models [10–14]. In our previous study, we found that M2 macrophages are involved in the pathogenesis of acute tubulointerstitial lesions in glomerulonephritis patients with crescents, acute tubulointerstitial injury of acute tubular necrosis and acute interstitial nephritis patients with good prognosis [15,16]. In this study, CD163-positive macrophages also accumulated in areas of acute tubular injury and positively correlated with urinary NAG and β2-microglobulin, which implied CD163-positive macrophages may be involved in acute tubular injury in pSS. We also found that HIF-1α was observed in acute renal injury and was co-localized with M2 macrophage. HIF-1α is considered as a key regulator of oxygen homeostasis, which mediates proximal tubule cells survival and recovery in response to ischemia-reperfusion injury *in vitro* and *in vivo* through the induction of a variety of oxygen regulated and renoprotective genes [17,18]. It suggests that HIF-1α and M2 macrophage might be involved in the repair of acute tubular injury of SS.

During follow-up of one year, six patients with pSS and acute tubular injury acquired improved renal function on therapy of middle dose of steroid and total glucosides of peony. The remaining four patients with pSS had stable renal function. Prompt therapy and reversible acute tubular injury might be the reason why the renal function was stable in the pSS patients.

Compared with CIN, patients with pSS had lower urinary osmotic pressure. Urinary osmotic pressure positively correlated to CD163 positive cells and CD68 positive cells, which suggest that both acute and chronic tubular injury contributes to the decrease of renal concentration function. We observed more macrophages in tubulointerstitial area of hypokalemia patients, which suggest that hypokalemia could lead to tubular injury and correct it could slow the progression of chronic tubulointerstitial lesions. Further research is needed to ascertain whether interventions to increase the expression of CD163-positive cells would help to resolve acute tubulointerstitial lesions in animal models of pSS.

In summary, M2 macrophages and HIF-1α are involved in acute tubular injury in patients with primary Sjogren's disease. Early intervention can improve renal function of tubulointerstitial disease secondary to primary Sjogren's disease.
